# Reflecting on chiral chimeric cancer and microbes: ambidextrous metabolism—the real danger?

**DOI:** 10.3389/fcell.2025.1586481

**Published:** 2025-09-11

**Authors:** Chika Edward Uzoigwe

**Affiliations:** Department of Science, Medicine and Surgery, NHS England, London, United Kingdom

**Keywords:** mirror life, ambidextrous life, homochirality, heterochirality, dual chirality, ambidextrous metabolism

## Abstract

The advent of genetic technologies requires consideration of neo-microbes. Attention must first be given to mirror life-forms that exhibit chirality discordant to that prevalent in nature. It is important to understand the merits and challenges faced by “contra-lateralized” organisms. The hypothesis here is that such organisms would suffer almost insuperable disadvantages. Their energy transduction mechanisms would result in ATP hydrolysis. They would be vulnerable to both innate achiral and acquired bi-chiral host immunity, but their defense and virulence mechanisms would exhibit heterodox chirality and thus be ineffectual. They would be savagely out-competed by commensals. It is hypothesized that the greatest utility and threat is from “ambidextrous” species that exhibit chiral chimerism/chiral duality. Different functions would be executed by effectors of varying chirality which may be inducible or facultative. Such microbiota would show predominantly conventional biochemical “lateralization”. However, few strategic functions would exhibit non-conformative chirality. One of the most significant dangers and potential benefits is the sheer unpredictability of the host response to counter-chiral antigens/molecules. Some synthetic enantiomers are an order of magnitude more active than their stereoisomer. Exceptional hazards thus lie in chiral chimeras that may elicit a hyper-exuberant immune reaction and shield themselves from that immune response by deploying a “cloaking” device in the form of a contra-chiral cell wall. As proof of this principle, cancer, which frequently overwhelms hosts, shows biochemical ambidexterity with bio-affinity for both L-glucose and D-glucose uptake and potentially also exploits D-amino acids for protein synthesis. Intriguingly, organisms of varying sophistication exhibit varying degrees of chiral duality. Hetero-chiral D-alanine and L-galactose derivates (fucose) and conjugate enzymes, for example, are bio-molecular protagonists. Extreme caution is required with such stereo-diverse agents, especially given that their chiral plasticity would be transmissible via plasmids or recombination, unlike obligate “mirror” species. However, effective regulation is fraught with obstacles as non-canonical chiral and bi-chiral enzymes and molecules already exist in nature, serving roles germane to species’ survival. The fundamental question is whether there is a need for a critical threshold for the heterochiral metabolic enrichment of organisms beyond which a tangible hazard subsists.

## Introduction

Heterochiral microbiota are a powerful but agathokakological invention which offer great opportunities but potentially pose a significant hazard (Adamala et al., 2024). On 12 June 2025, the UK Government published a research and analysis guidance piece on “Mirror Life” (https://www.gov.uk/government/publications/mirror-life/mirror-life.) that highlighted the potential advantages and future dangers of research in this field. The US government (https://www.congress.gov/crs-product/IF12883) and influential thinktank RAND issued similar publications in January 2025 and March 2025, respectively. The latter advocated a moratorium on research until the magnitude of the threat had been definitively determined.

The creation of counter-lateralized species may not necessarily have a sinister motivation. Non-conformative chiral bioagents can be exploited as a means of drug or bio-vaccine delivery. Such lifeforms may directly produce beneficial microbial effects. For example, their deviant biochemical “lateralization” may circumvent some of the challenges faced by viral vector vaccines such as immunity to the vector itself, which results in the destruction of the vector before it deploys its cargo (Wang et al., 2023). For example, the ChAdOx1 adenoviral vector is derived from the Y5 adenovirus strain, for which 9% (95%: CI: 3%–20%) of adults in Gambia carry neutralizing antibodies but 0% (95% CI: 0%–4%) (Dicks et al., 2012) of UK adults do. Whichever vector of conventional chirality is selected there will be a cohort that expresses the highest bracket of neutralising antibody titre, thus introducing inequity into an invention designed to foster health equity. This would not arise with a neo-chiral viral vector to which the world is immunologically naive. It is unthinkable that “mirror biomedicines” could potentially not only be therapeutically more efficacious but also combat health inequality. Furthermore, such life-forms could act as a bio-factory for an-immunogenic, protease-resistant drug agonist and antagonists. Uppalapati et al. (2016) created a heterochiral (D-) protein antagonist of VEGF-A (vascular endothelial growth factor- A) of considerable durability due to low immunogenicity and resistance to proteolysis. Their most difficult process was the chemical synthesis of the compound, which was facilitated by contra-enantiomeric biota.

The potential benefits notwithstanding, given the recent devastating global toll of unexpected pathogens, the dangers also inherent to nouveau-enantiomeric lifeforms must not be underestimated. Even if motivations were singularly benevolent, it is necessary to fully understand the challenges faced by heterochiral species so as to engage the most fruitful avenues of research. Present homochirality is so fundamentally engrained in life since its inception, and indeed in its prebiotic forms, such that new organisms, not conforming to this chiral blueprint, will face an almost insurmountable disadvantage. Herein I show that chiro-divergent bacteria would be enthalpically non-viable, exceptionally vulnerable to host immunity, and themselves anodyne and savagely outcompeted by commensals (Table 1). We conclude that the most fruitful and paradoxically most risky approach lies not with the exploitation of mirror life but of “ambidextrous” pathogens that exhibit chiral chimerism. They deploy different chiral effectors for different functions. This has the potential to exploit and subvert the host (Table 1). As proof of concept, there already exist varying degrees of biochemical dual chirality that are physiologically and pathologically operative in nature for such purposes. This renders the prospect of comprehensive and incisive regulation ambitious. It also mandates consideration of putative methods of addressing any challenges posed by ambidextrous and dual chirality organisms. There is a gap in the literature in this area.

**TABLE 1:** Summary of challenges of heterochiral and ambidextrous species.

Challenge	Homochiral	Heterochiral	Ambidextrous
Energy Generation: ATP synthase	Antegrade proton flow generates ATP	Antegrade proton flow degenerates ATP to ADP to Phosphate	Successful strains will exploit homochiral blueprint
Electrochemical Factors			
3 Na+/2 K+ co-transporter	Exports 3 Na+ and Imports 2 K+. Maintaining negative membrane potential. K egress potentiates negative membrane potential	Imports 3 Na+ and exports 2 K+. Could maintain positive membrane potential. Na egress would dissipate membrane potential	Successful strains will exploit homochiral blueprint
Membrane Potential	Negative membrane potential. Potentiates microbial aggregation and biofilm. In water like-negative charge micro-particles attract	Would likely maintain negative membrane potential. Were they to have a positive membrane potential this would resist microbial aggregation biofilm. In water like-positive charge micro-particles repel	Successful strains will be negative but may be positive as heterochiral
Immunity			
Innate Immunity			
PF4	Attracted to negative charged species	Attractive to negative species and even negative membrane phospholipids. May evade with positive membrane potential	May evade with membrane potential
Complement	Vulnerable to all limbs of complement (Classical, Lectin & Alternative) but produces euchiral mediators that could block complement	Likely resistant to Classical and Lectin pathways of Complement Vulnerable to a number of elements including the achiral for example, Alternative Pathway C3 thiol binding to hydroxyl species. Their defense armamentarium is heterochiral and hence counter-chiral to complement and unable to activate	Likely resistant to Classical and Lectin pathways of Complement. Vulnerable to the alternative pathways but has the potential to exploit cyn-chrial enzyme inhibitors of Complement
Acquired Immunity			
Antibody mediated	Vulnerable	Certain chiral motifs may be invisible. However L-fucose is immunogenic	Will achieve antibody invisibility by a combination of eu- and counter-chiral motifs

The almost insuperable challenges faced by exclusively counter-lateralized organisms are addressed first.

## Thermodynamic viability

Chiro-discordant microbes are potentially energetically non-viable. One need only look for examples in the most fundamental and empiric processes, such as those related to energy transductions. ATP synthase is contingent upon a rotatory mechanism whereby the transit of three protons generates one molecule of ATP by sequential 120^o^ third-rotations. This is a chiral process with a chiral enzyme and organism. If the chirality of ATP synthase is reversed, standard proton migration would result in the degradation of ATP to ADP and phosphate (Nesci et al., 2015) and would be deleterious to the hetero-enantiomeric pathogen. An efflux of protons would be required for ATP generation which would potentially require a mirror microbe to reverse its membrane potential, such that it be positive in polarity. While this may not be insuperable, it is exceedingly challenging. The negative membrane potential is preserved by a number of factors, the majority, if not all, of which are non-chiral and dependent upon the fundamental nature of the most abundant and diminutive ions (Benarroch and Asally, 2020). The first is the selective permeability of the membranes to ions. This is a function of the fundamental physical properties of the most prevalent and bioactive ions on earth such as sodium, chloride, potassium, and calcium, all which are achiral. The second is the presence of ion transporters and channels. These, unlike ATPases, lack a chiral-contingent rotatory mechanism; hence any chirality they do exhibit does not necessarily affect the direction of movement of achiral metallic ions. Finally, any asymmetry in the electrical charges of membrane lipids on either side of the membrane (Ohki phenomenon) may play a role (Hughes, 2024), which is again unrelated to chirality. Even if the polarity of the ATPase was reversed, this would merely result in heterochiral ATP being created and released outside of the anti-enantiomeric pathogen. Simply put, membrane potential is not dependent upon chirality, but ATP generation is. This mismatch poses an existential conundrum for mirror lifeforms in how to choreograph proton migration down an achiral electrochemical gradient with chiral ATP synthesis.

ATP synthases are amongst the most efficient enzymes, with efficiencies of 79% to 217% reported (Toyabe et al., 2011; Silverstein, 2024). If micro-biota, exhibiting non-normative chirality, are divested of this enzyme and must rely on chemical and/or glycolytic rather than biological reactions for ATP generation, their enthalpic survival would be critically jeopardised. Further microbes and pathogens, in particular, expend vast amounts of energy on maintaining the electrochemical gradient and immune evasion (Acosta and Alonzo, 2023). Non-canonical chiral organisms are thus hugely disadvantaged in these energetically demanding feats.

## Electro-chemical chiral conundrum

The Na+/K + ATPase counter-transporter sequesters anywhere from 20% to 70% of a cell’s ATP (Mobashe et al., 2000). Canonical Na+/K + ATP synthase imports two potassium ions for every three sodium ions exported. Theoretically, mirror versions of Na/K-ATPase may import three sodium ions for every two potassium ions extruded. This would accumulate sodium and tend towards a positive membrane potential. However, the egress of sodium would dissipate the membrane potential. This may be compared to homochiral Na/K-ATPase. The accumulation of two potassium ions for three sodium ions results in a negative membrane potential in addition to accumulation of potassium. The diffusion of potassium down the concentration gradient forms the negative potential. As such, a counter chiral Na+/K + -ATPase would be thermodynamically unfavorable. A positive membrane potential is also resisted by the asymmetric negative charge on the heads of phospholipids.

Challenges exist with a reverse Na+/K + -ATPase polarity increase, and new challenges emerge even if the cell were able to achieve the positive membrane potential. Evidence shows that negatively charged microparticles, the size of microbiota, actually attract each other and aggregate in aqueous milieux (Wang et al., 2024). This is due to the polarity of water molecules which change their orientation as two negatively charged particles approach; releasing energy and rendering like-negatively charge attraction enthalpically favourable. This may facilitate cellular aggregation and biofilm formation of microbiota. Similarly, like-positively charged species repel each other in aquatic environments but attract in alcohol. However, evolution very much favors water over alcohol.

Even assuming that chiro-deviant organisms were capable of maintaining enthalpic integrity, they must then contend with the sophisticated counter-biotic attack of host immunity honed over billions of years.

## Immune vulnerability

### Achiral innate immunity

Non-canonical chiral lateralization alone would not protect mirror life-forms from host immune attack. Even the most rudimentary immune systems, and especially human immunity, have a number of modes of attack—specifically for bacterial enantiomers. These are part of what I term the “ontological” arsenal, as they are effective simply because a pathogen exists. Synchiral pathogens deploy chiral immune evasion mechanisms, such as chiral-specific proteases and blockers, which are not at the disposal of chiro-divergent species (Heggi et al., 2024). Simply put, hosts intentionally employ non-chiral generic antimicrobial strategies, against which microbes must use normo-chiral mediators to evade. If the latter do not, they are eradicated and cannot elicit an infection (Uppalapati et al., 2016). The immune system operates certain mediators which are chiral in structure but function in an exclusively achiral manner. The only way in which microbes can and do function against attack from these must necessarily be via homochiral proteases and blockers. If they exhibit unorthodox chirality, they fail this test.

Consider first the alternative pathway of the complement system (Mastellos et al., 2024). This is healthy ontological immunity—a contra-biotic system that is perpetually active. Host cells themselves are only protected from attack as they actively express factor H and I which disarm this limb of immunity and act as a “seal of self-ness” (seal of *ipse*). Factors H and I are essentially antithetical (Meri, 2016). Heterodox chiral pathogens, on the other hand, would be a prime target. The complement assault molecule C3b binds in an intentionally achiral manner to pathogen surfaces. It deploys a highly reactive thiol species to bind to both hydroxyl and amine moieties—the elements of life. To survive in a host, bacteria must evade this system. They achieve this by targeting C3b and other antecedent and descendent effectors with enzymes and inhibitors which are chiral (Heggi et al., 2024; Serruto et al., 2010). Hence, this host achiral attack can only be undone by a chiral defence. The host has complete advantage over the mirror microbe.

Consider now PF4 (platelet derived factor 4). This is an element of innate immunity. It binds negative polyanionic species irrespective of chirality, resulting in a conformational change in PF4 (Greinacher, 2015; Ngo et al., 2023). Antibodies then recognize this conformationally transformed PF4, even if the antigen binding PF4 is entirely unrecognizable. This potentiates the host’s recognition of novel antigens when represented unbound to PF4. Indeed, the PF4 mechanism is so sensitive that it can be triggered by anodyne antigens such as heparin, resulting in heparin-induced thrombocytopenia (HIT), by vaccines in vaccine induced thrombotic thrombocytopenia (VITT), and even by self-antigenic polyanions to which the host’s own acquired immunity has established tolerance (Nguyen et al., 2017). The immune system’s subsequent most potent antimicrobials are both non-chiral and innate—notably reactive oxygen species in the form of nitric oxide, free radicals, and peroxide moieties (Manoharan et al., 2024). Neither their D-amino acids nor their L-saccharides can save a mirror microbe from this immunological onslaught if it has no defense. https://www.sciencedirect.com/science/article/abs/pii/S1471490623002648.

#### Biological effects of the loss of homochirality in a multicellular organism

##### cGAS-diSTINGuishing between enantiomers

Canonical natural DNA comprises D-pentose residues. This is the R-enantiomer, which is a right-handed (clockwise turns along axis) double-helix. It is most commonly B-DNA. Non-canonical DNA L-enantiomer is a sinister (left-) handed double helix. Its constituent pentose-monomers are the L-isomer. One would anticipate that mirror life DNA would be a left-handed helix and thus evade this arm of innate immunity. However, canonical R-DNA, even with D-pentose residues, also exists in nature as a left-handed helix. This is termed “Z-DNA”. Human hosts have an armada of anti-microbial mediators that bind left-handed helix (Z-DNA) and thus will likely detect heterochiral invaders. The cGAS-STING axis, against free-cellular double-stranded DNA, is one of the most atavistic and yet potent pathways of innate immunity. It may well have its origins amongst the first primordial components of the anti-viral arsenal of bacteria (Vance, 2024). The axis persists today in higher species, including humans, seamlessly integrated into our sophisticated immune systems. cGAS (cyclic GMP–AMP synthase) binds free cellular double-stranded DNA. It then dimerizes and stimulates STING (stimulator of interferon genes). Its targets are alien viral and/or bacterial DNA. Interferon is potently anti-microbial and protects adjacent host cells from attack. cGAS exhibits helical promiscuity and will bind both right- (B-) and left-handed helical (Z-) DNA. The aptly-named Z-DNA binding protein 1 (ZBP1) also exhibits avidity for both (heterochiral left-handed) Z-DNA and Z-RNA (Herbert, 2019). ZBP elicits dense cellular arrest, resulting in PANoptosis. Host immunity appears to expect the existential threat posed by heterochiral life. The response mounted to the heterochiral helix of Z-DNA is decisive—cGAS-STING and ZBP act cooperatively to eliminate sinister (left) helical DNA (Z-DNA) (Chen and Kanneganti, 2023). DNA from mirror microbia is likely to be very susceptible.

Certain microbia do generate specific 2′,3′-cGAMP nucleases that degrade cyclic GMP-AMP, thereby mitigating cGAS immunity (Decout and Ablasser, 2019). However, this is only at the disposal of chiro-conformative hosts. cGAS is an ambichiral immunity and hence targets mirror and homochiral life. However, it can only be neutralized by factors that exist as an enantiomeric match.

### Bichiral acquired immunity: Double (chiral) agent: IgG3?

The suggestion that mirror life would show complete resistance to antibodies from ortho-chiral (homochiral) hosts may be overstated. Evidence suggests that non-canonical chiral amino acids prime host immunity (Suzuki et al., 2021). Bacterial heterochiral amino acids promote the growth and survival of gut macrophages and B cells, effectively acting as counterchiral signals to alert this aspect of immunity to bacterial presence. Van Ree et al. (2000) have shown that the pollen allergen Lol pX1 triggers the generation of IgE which specifically binds to the residue fucose—L-6 deoxy-galactose—and thus a heterodox chiral derivative of D-galactose. It cannot be assumed that non-canonical chirality bio-molecules are not necessarily immunologically inert.

In murine experiments, Benkirane et al. (1993) converted terminal 7 amino acids from histone H3 from D- to L-amino acids. They found that mice produced exuberant antibody responses to this D heptad of amino acids. These antibodies also cross-reacted with their sister (sinister) L-enantiomer heptad and L-enantiomer H3-parent. Similarly, antibodies directed to the L-terminal H3 heptad and H3 also recognized the D-analogue heptad. The most intriguing element of this study was that it was exclusively and consistently IgG3 alone that was capable of bi-chiral affinity; it was not observed in any other IgG class. This strongly suggests an evolved trait to a threat that is deemed novel to science but arose by natural selection hundreds of millions of years ago. IgG3 may have the role of targeting chiro-divergent pathogens. The most remarkable aspect of this is that L-amino acids generate right-handed (clockwise along axis) ɑ-helices and D-amino acids left-handedly (anti-clockwise along axis), yet IgG3 recognizes both. Despite the sophistication of the system, IgG3 accounts for only 7%–10% of IgG in both human and murine immune systems (Vidarsson et al., 2014; Sarvas et al., 1983). However, it is both an ultra-high affinity ligand and the most efficacious at recruiting other aspects of immunity and is particularly complementary (Damelang et al., 2019; Abendstein et al., 2023). This agrees with its chiral plurality. It would almost seem as though IgG3 has evolved specifically for epitopes that evade all other limbs of the immunity by, for example, chiral molecular subterfuge. When these antigens are detected by IgG3, the latter then recruits all other immune elements the pathogen had thought it had evaded. The other IgG subclasses thus appear to work in parallel or concert with other immune axes, however IgG3 acts a focal hub or epicentre of an immune nexus for the most latent and elusive antigens such as those exhibiting heterochirality. Hence neo-chiral antigens, initially only susceptible to “ontological” achiral complement C3b assault, on binding to IgG3 are then vulnerable to the Classical Complement pathway.

The trans-chiral immune potency of IgG3 is also demonstrated, paradoxically, by its ability to bind pathological counter-chiral antigens. Systemic Lupus Erythematosus (SLE) is an autoimmune condition characterised by antibodies against self double-stranded DNA. As previously stated, B or -Right-handed helical double stranded-DNA is the physiological form. The DNA found in SLE is Z or left-handed DNA (Lafer et al 1983). This would also be the predominant form in mirror microbes as they would comprise an L- rather than D-pentose spine. Now a cardinal subclass of self-antibodies against this Z-DNA in SLE, is, as one would anticipate, IgG3 (Devey et al., 1988).

### Host response: Sufficient or subliminal?

As further evidence of the antigenic enantiomeric inclusivity of human immunity, Xu et al. demonstrated that human immunity shows responsiveness to nanoparticles of both L and D chirality. This response is asymmetric, however, with a heightened reaction to L-lateralised nano-particles (Hooftman and O'Neill, 2022; Xu et al., 2022). Heterodox chiral (that is D-) proteins show low immunogenicity as compared to D-oligo-peptides. It has been suggested that this is attributable to the fact that the latter require little immunological pre-processing prior to entering immunity pathways, which is not the same with macromolecular D-proteins. The D-amide bonds may restrict immune sorting. This raises an intriguing speculation as to whether superantigens from chiro-divergent species would be noxious to hosts. Superantigens are microbial antigens that elicit a massive disproportionate immune response recruiting various limbs of immunity (Tuffs et al., 2024). They are modestly sized single-chain polypeptides that, by design, avoid processing and presentation in antigen-presenting cells (APC). Antigens are presented by an APC to CD4+/CD8+ T-cells, creating an immune synapse. Superantigens keep the APC and T cells constitutively engaged and trigger proliferative immune cascades and nexus. If heterochiral oligo-peptides are immunogenic due to their diminutive size and lack of need for APC processing, superantigen from mirror-life may equally exhibit super-immunogenicity. However the composite evidence suggests they would be much more likely sub-rather than superantigens. Classical chirality superantigens bind in such an intimate, sophisticated, and intricate manner to both the APC Major Histocompatibility Complex II receptor (MHC II) and CD4^+^ and/or CD8^+^ receptor of T cells. It seems highly unlikely, if not impossible, that their enantiomeric anlage would exhibit binding of similar affinity. This highlights the fact that chiro-divergent species would simply lack the arsenal to harm the host.

Host response must be tempered against the pathogenicity of counter-enantiomeric microbes. One must contest the notion that evasion of the host immunity will necessarily result in lethal infection. The reason for this is that for the vast majority of pathogens, if not all, their most lethal virulence factors are chiral and depend upon the host physiological and/or immune machinery recognition for their harmful effects (Leitão, 2020; Narciso et al., 2025). Simply put, no pathogen can be invulnerable to immunity and yet lethal to host. Consider *Staphylococcus aureus* as a classic example. Virulence factors include enzymes coagulase, hyalauridase, and deoxyribonulcease. The heterochiral forms would be ineffectual in a normochiral host. *S. aureus* also deploys enantiomer specific “super-antigens” in the form of the hazardous enterotoxins and exfoliative toxins. Another potent piece of armamentarium is the pathogen’s Type II secretion system, which allows the bulk secretion toxins into host cells (Taj and Chattopadhyay, 2024). This comprises ATPase moieties which would simply be non-functional with heterodox chirality, due to the need for katadromic (retrograde) proton flow for activation as discussed above.

## Stereo-isomeric stochasticity

The discussion on mirror life, especially in the arena of immunology, must be tempered with the caveat that the physiological response to enantiomeric biomolecules is unpredictable, inextrapolable, protean, and dependent upon organ systems. Different players within the same homeostatic nexus exhibit differing degrees of enantiomer selectivity and permissiveness. This can lead to harm. D-glucose is physiologically occurring. L-glucose is exceptionally rare in nature (Yamada et al., 2017). The human physiology cannot metabolize L-glucose. Nonetheless, this L-monosaccharide is indistinguishably as sweet as D-glucose. Further, it can stimulate the pancreas to secrete insulin (Malaisse, 2014). An un-metabolizable carbohydrate stimulates insulin release, which is typically released in response to carbohydrates amenable to host metabolism. This can potentially result in hypoglycemia. However, it could also be co-opted to treat diabetes.

Mal-chiral bio-forms may even interrupt and hijack therapeutic pathways. NNC2215 is a novel synthetic glucose-responsive insulin, purposed to treat diabetes. When D-glucose levels are high it is bio-active and promotes glucose uptake; when levels are low it is deactivated (Hoeg-Jensen et al., 2024). However, it is also activated by the hetero-chiral L-glucose. Due to the chiral mismatch in response, cells will not uptake L-glucose but only D-glucose. Hence, a bio-vector/agent generating L-glucose and/or other stimulagogue/analogue will activate NC2215, resulting in D-glucose sequestration from the blood into insulinotropic cells. However, the L-glucose stimulant is never cleared from the blood but perpetually activates NC2215 until D-glucose is totally eliminated, rendering a healthy medication almost lethal.

A host of selective serotonin inhibitors (SSRIs) have been developed to treat depression. Surprisingly, in most cases, both enantiomers are bio-active, however one much more so than the other. This is most striking in the case of citalopram, where one enantiomer has 30 times the inhibitory effect of the other (Budău et al., 2017). Extrapolation necessarily must be made to counter-lateralized microbiota and their metabolism. A non-conformative chiral antigen may thus become an ultra-antigen eliciting the most hyper-exuberant immune responses, with radical sequel. Glucagon, as a further example, increases blood glucose and is released as a result of fasting or a high-protein meal (Wewer Albrechtsen et al., 2023). It is wholly unclear and unpredictable which, if any, D-amino acids would trigger glucagon release. Following the example of citalopram, a neo counter-chiral D-amino acid could conceivably be 30 times more potent that L-enantiomer analogue at precipitating glucagon release.

Consider also the enantiomer polarities of penicillamine. The D-stereoisomer is a therapeutic agent used in the treatment of rheumatoid arthritis and cuprate metabolic disease (Wilson’s Disease). L-enantiomer is toxic, competitively inhibiting vitamin B6 (pyridoxine) (Kuchinskas et al., 1957).

## Commensal competition

It is suggested that pathogens with non-conformative chirality would essentially, at most, be commensals. If they exhibited any pathogenicity they would be eradicated, predominantly by achiral methods to which their heterochiral defenses would be ineffectual. Commensals regularly access the blood. Immunity consistently exhibits a permissive approach to such species (Maier et al., 2024; Tan et al., 2023). Even if the mirror microbes would attempt to colonize new niches, they would suffer from competition with homochiral pathogens. Resource sequestration by commensals is a means of controlling pathogenic bacteria (Mobashe et al., 2000; Pamer, , 2024). A maladapted heterochiral pathogen, reliant on additionally enzymes to metabolize D-glucose, would be hugely disadvantaged and out-competed. Those with a very narrow exclusively achiral nutritional repertoire would suffer a similar fate.

Hosts deploy resource sequestration tactics which are inescapable for chiro-divergent pathogens. Essential trace nutrients, such as iron, zinc, copper, and manganese, are bound in chiral organo-metallic complexes that are impenetrable for mirror microbes, which must overcome both high affinity binding of ion-organic complexes and the chiral mismatch (Murdoch and Skaar, 2022). Further, pathogens are reliant upon biofilms and quorum sensing to improve population efficiency and provide some resistance to both innate and acquired immunity. Unorthodox chiral microbia could not exploit established biofilms as their surface receptors would not recognize the chiral biofilm biomolecules (Mukherjee and Bassler, 2019). They would be an exposed pioneer dependent upon creation of its own biofilm.

## Mirror microbe mass menace?

The “invisible microbial mass” pathogenesis hypothesis suggests that mirror life-forms would be immunologically undetectable by the host and thus latently foment to a “heterochiral microbial tumor” sequestering host resources and ultimately causing a mass effect or sterile abscess. This is not entirely persuasive. Gut commensals with a cornucopia of trophic resources, syn-chirally matched to the host and surrounded by an indifferent immunity, do not grow in an unregulated fashion, dampening the activity of the gut and bowel. “Heterochiral microbial tumors” have been likened, in pathogenicity, to cancers. However, they would lack the eu-chiral signaling repertoire to effect angiogenesis to support this macro-structure. Neither would the host be overwhelmed in their demise from their heterochiral bio-load in any putative “mirror microbe tumor lysis syndrome”. Studies show that the mammalian body is adept at handling a chiro-diverse amino acid load. Gonda et al. observed that D-amino acids are present at modest but appreciable levels in human plasma but eliminated and enriched in the urine. The D/L-amino acid ratio (normo-chiral/non-normative) was 1:1,000 in blood but 1:5 in the urine and feces (Gonda et al., 2023). Indeed, gut fauna in mammals so frequently racemase L-amino acids to their D-counterparts, generating and employing the D enantiomeric anlage D-serine, D-aspartate, D-glutamate, D-alanine, and D-proline that species have developed specific adaptations for such phenomena (Sasabe et al., 2025). In all stages of amino acid metabolism, D-amino acids can be inadvertently implicated instead of the enantiomerically dominant L-chiro-form. Cellular processes detect these chiral errors and reverse and/or substitute with the “correct” stereoisomer. The process has limits. Drosophila exposed to a D-amino acid hyper-enriched environment suffered harm characterized by a higher tumor incidence and truncated longevity (Banreti et al., 2022). Protein-l-isoaspartate (d-aspartate) O-methyltransferase (Pimt) is germane to homochiral homeostasis in the face of the abundant heterochiral load. It specifically recognizes and converts d-aspartyl to orho-chiral l-aspartatyl.

One must conclude that counter-chiral amino acids are far from biochemically invisible to host molecular machinery notwithstanding the very strong dominance of one enantiomer over another.

## Ambidextrous microbia

Given the grave limitations of mirror life, possibly the most sinister class of bacteria would be chiral chimeras or “ambidextrous” microbes where subcellular function dictates chirality. Such pathogens could evade immunity with a non-conformative chiral membrane and wall but deploy defense mechanisms and virulence factors that were normochiral. They would exploit homochiral metabolites and biofilms. This may manifest in a number of guises including facultative and or inducible chiro-divergence. Indeed, in their most sinister guises, chiral divergent evasive maneuvers may be triggered by a stress-response to immune attack. Obligate mirror microbes lack metabolic machinery to be a sustainable threat. The threat level is amplified by the fact that this virulence factor of chiral plasticity would be transmissible to other microbes via plasmids or recombination events. This would be impossible for obligate mirror lifeforms. The advent of AI potentiates our ability to realize the most ruthless metabolic chiral combinations of ambidextrous life.

## Chiral combat: current ecosystem ambi-chirality

While homochirality is pre-eminent, it is far from the exclusive bio-physiological motif. Many organism systems remain significantly lateralized but nonetheless exhibit a degree of dual chirality and ambidexterity.

### Bilingual DNA binding and bichiral chaperone proteins

Weil‐Ktorza et al. reported the identification of an ambidextrous protein exhibiting high affinity binding to both canonical chiral (D-) and non-canonical (L-) DNA (Weil‐Ktorza et al., 2023). Folding chaperone proteins can also martial both D and L amino acids chains (Weinstock et al., 2014). This led the authors, amongst others, to speculate the origin of such chiral versatility. It has been assumed that this is an atavistic or primitive state (Callaway, 2025) but it may equally be derived. A distinct possibility is that counter-chiral bio-molecules may have been perpetually in existence and in consistent evolution but their capabilities are not perceived for the simple reason that there is no physiological challenge, threat, or occasion for them to be manifest. Heterodox chirality may be redundant but pose no real harm. There is thus no specific reason why dual chiral enzymes should be deselected. One must wonder if there is a paucity of bio-molecular ambidexterity or if there has not been the occasion or the initiative to assay natural occurring enzymes for this phenotype. This leads to fundamental questions as to the provenance of “contingent homochirality”. For example, how did the RNA, encoding enzymes, “know” that glucose is predominantly D-glucose or “know” that amino acids, in principal, exist in the L-chiral blueprint and DNA in the D motif. Were dual chiral dexterity the biological norm from the start then there is no reason this should have been deselected. Perhaps we have hitherto been too incurious in the domain of ambidextrous metabolism.

### Latent ambidexterity

As discussed above, S-citalopram is 30-fold more efficient than the R-enantiomer, indicative nonetheless of some degree of ambidexterity in serotonin uptake enzymes (Koldsø et al., 2010). However, this only became apparent with the creation of citalopram. Otherwise it would be latent and undetected. Similarly, synthetic L-glucose is sweet like chiro-orthodox (homochiral) D-glucose and can stimulate insulin release (Malaisse, 2014). Again, this is demonstrative of a dual chirality demonstrable only with the advent of human pharmacopoeia.

### Dual chiral saccharide metabolism

Working in favor of ambidextrous chirality as an archaic rather than derived trait is the fact that possibly the first cohort of metabolic enzymes, the saccharides dehyrogenases, show chiral promiscuity: accepting counter-chiral L-glucose and L-xylose (Frazão et al., 2023). This is typified by D-threo-aldolase dehydrogenase of *Paraburkholderia caryophylli* that accepts L-glucose as a metabolic substrate.

Organisms actually exploit and selectively enrich counter-chiral substrates for their own ends in both defense and attack. Arabinose is a very common natural monosaccharide, however, unlike most other saccharides, in nature it is found almost exclusively as the L-enantiomer as opposed to the D-enantiomer. It is a cardinal component of plant cellulose cell walls. It thus has an architectural and protective role (Kumar et al., 2024). The polymer is physiologically robust but the “heterodox” L-arabinose monomer also renders it enzymatically resilient. One can only assume that L- and D-arabinose were formerly in enantiomeric equivalence or with D-arabinose dominance but the L-arabinose then enriched by natural selection. However, while saccharides have pleiotropic and versatile roles, the ubiquity of arabinose is due to the prevalence of cellulose rather than as a result of a broad and inclusive use of the L-saccharide *per se* in varying biochemical processes.

Similarly, L-fucose is 6-deoxy-L-galactose that opposes the chiral convention of D-saccharides’ biological supremacy. L-fucose is the seminal component of the seaweed protective polymer fucoidan (Kumar et al., 2024). Seaweed is notoriously exposed to hostile milieux that would facilitate enzymatic degradation of canonical polymer bonds–notably an UV-irradiated aquatic environment, replete with transition metals, halides, alkali, and alkali earth (calcium/magnesium) metal ions.

In addition to preserving cellular structural and physical integrity, fucose is also the guardian of metabolic signaling integrity. Fucosylation is a common post-transcription modification that refines and alters function (Schneider et al., 2017). As the bond is to a non-canonical enantiomer, it is almost invulnerable to conventionally chiral-degradative enzymes, thus resulting in an almost indelible molecular tag. Fucosyaltion modifies antibody function by potentiating antibody-mediated cytotoxic activity (Golay et al., 2022). It also a seminal component of the erythrocyte blood group glycocalyx, which may have its origin in helping the host distinguish self from pathogen antigens. Fucose moieties are at the terminus of all the ABO and Lewis blood group-specific epitopes. The acral (terminal) fucosylation in two distinct systems suggests this is more than coincidence. The heterodox L-saccharide bonds prevent degradation and thus impart an almost ineffaceable group or immune tag.

Most intriguingly, fucoidan also exemplifies the greatest hazard attendant to heterochiral molecules, namely their idiosyncratic and unpredictable effects. Fucoidan is a potent anti-coagulant, defunctioning the classical coagulation pathway (Chen et al., 2025). Significantly, deployment of the clotting cascade and the platelet recruitment is necessary for an effective immune response and controlling the dissemination of infection. Further, it suppresses the immune system (Vo, 2020; Yeh et al., 2022). It is clear that fucoidan variants, if appropriately enriched, could trigger a devastating immune suppressive-coagulopathic attack. This is potentiated by the fact that the non-canonical L-saccharide polymer would be resistant to degradation.

### D-Amino acids

#### Microbiota

The Earth’s chirally polarized metabolism gained some ambidexterity in order to co-opt heterochiral biomolecules predominantly for bio-architectural roles. A similar pattern is observed with “non-canonical” D-alanine, which is found frequently in nature. Similar to L-arabinose, D-alanine has a cardinal architectural role. It is a protagonist monomer in the constituent of bacterial cell walls, a peptidoglycan formed by repeating units of N-acetylglucosamine and N-acetylmuramic acid. The latter is appended counter-chiral oligo-peptide bridges comprising D-amino acids, including variously D-alanine and D-glutamate (Blanke, 2009). This heterochiral circumvallation would appear to serve the function of fending off expo-peptidases. In primordial coacervates, degradative enzymes would have only been imperfectly affiliated to and segregated by proto-cells (Cao S. et al., 2024). As suggested by the “patchwork model”, the first enzymes would have necessarily been promiscuous with regard to substrate (Leveson-Gower et al., 2019). Indeed, the inception of life may have been dependent upon this broad substrate repertoire of the earliest enzymes. If cellular compartmentalization, bio-polymerization, and durable signaling were to evolve, this would necessarily require the use of counter-conventional enantiomers to supplant chiro-conventional motifs recognized by the broad spectrum exo- and endo-peptidases; otherwise, cellular survival and function would be impossible. As proof of concept, the racemase enzyme essential for the conversion of L-alanine to D-alanine is essential to the virulence of a number of bacteria, notably in those in the oro-digestive tract (Wei et al., 2016).

It was previously speculated that ambidextrous microbes would deploy syn-chiral effectors to exploit the host but conceal itself from host immunity with a counter-chiral cell membrane. This is more than speculation but almost a distinct reality. The cell membranes of Group A *Streptococcus* (GAS) comprise techoic acids that impart the membrane with a negative charge via the carboxylates species. However, Kristian et al. showed that GAS also carry the gene, dlt-A, which causes the non-canonical d-alanylation of the cell membrane techoic acids (Kristian et al., 2005). The authors showed that this process converts the membrane from a negative to positive charge and directly enables GAS immune evasion and invasiveness. It specifically increases GAS resistance to cationic antimicrobial peptides, lysozyme, and neutrophil attack. Further, the inclusion of the heterochiral residues in D-alanine preserves the cloak from peptidase attack.

In summary, the presence of bilingual DNA binding proteins (with affinity for both R and L DNA), ambidextrous chaperone proteins, and genome encoding for the addition of D-alanine to the cell wall all point to a natural order adept at manipulating chirality to its own ends. One must consider if ambidextrous life is indeed not inevitable. Over millennia, as microbia learn to court more sophisticated immune systems, it seems conceivable they would have increasingly had recourse to heterochirality as an additional element to their armamentarium. It is postulated here that the advent of antibiotics reframed the host-pathogen relationship such that the primary threat to survival became homochiral antimicrobials agents, thus skewing microbial defense mechanisms toward antibiotic resistance over phenotypes that enable the host to subdue or circumnavigate host immunity. Antibiotics may have averted the advent of ambidextrous microbiota.

#### Megabiota

D-alanine and other D-amino acids have also been observed in a number of other higher order species including mollusks, spiders, and even the platypus (Torres et al., 2006). In the latter they are a key component of the venom. This is one of the few examples of counter-chirality used in a physiological aggressive fashion. It is thought that the incorporation of D-alanine prevents or retards degradation of the venom by the peptidases of the victim. There is thus the selective use of hetero-chirality for this purpose.

The sophistication, precision, and finesse with which evolution employs counter-chirality is remarkable. D-serine is a neurotransmitter even in humans (Wolosker, 2006; Wolosker and Balu, 2020). This may initially seem idiosyncratic. However, D-serine is a ligand for the NMDA (N-methyl-D-aspartate) receptor, involved in plasticity learning and memory. Canonical chirality L-serine is too short-lived to be a neurotransmitter. It would be degraded or sequestered in protein polymerization, hence the use of metabolism-resistant D-serine. Indeed, physiological enrichment of D-serine is quite remarkable. Up to 30% of serine in the forebrain exists as the D- (heterochiral) enantiomer, however only 1%–2% exists in the remainder of the body (Krishnan and Billups, 2023). Within the kidney there exist specific transporters for heterochiral amino acids, including D-serine. These transport heterologous chirality amino acids against concentration gradients into cells (Kimura et al., 2023). Even more spectacular than this, D-alanine has been shown to be generated in the gut of mice following action of gut commensal and thence act as a central neurotransmitter and part of the ethereal brain-gut axis (Lee et al., 2020; Qiu et al., 2023). The brain gut nexus has been implicated in a number of behaviors including acedia and willingness to exercise (Agirman and Hsiao, 2022).

It is clear that it is not so much thermodynamic constraints that limit ambidexterity but rather ambidextrous metabolism that would limit life itself. Lateralization may thus have been essential and germane to the evolution of sophisticated life. Were all enzyme ambidextrous, then all proteins and signaling molecules would be vulnerable. The protective layers of seaweed (fucoidan) and plants (hemicellulose) would be susceptive and digestible as starch and bread. Cellular segregation and protection critical for evolution would be impeded or totally arrested. Intricate and precision neuronal signaling would be adumbrated and blunted. Fucosylation would no longer be an indelible durable signaling but instead be a transient notice. Hence, sophisticated life may have only occurred because of biochemical lateralization, enabling the exploitation of counter chirality for specific cellular and structural functions.

### Chirality a la carte

The notion that hetero-chiral biomolecules are totally alien, unpalatable, and metabolically incompatible is far removed from organ systems actually adapted to a dual-chiral universe. Subcellular systems manipulate contra-chiral biomolecules when it is expedient to do so. α-Methylacyl-CoA racemase converts (2R)-2-methylacyl-CoA to its enantiomer (2S)-2-methylacyl-CoA (Lloyd et al., 2008). The latter is amenable to β oxidation, involved in the metabolism of fats, while the former is not due to chiral mismatch. Hence, this racemase increases the metabolic and trophic repertoire of the host. Intriguingly, α-Methylacyl-CoA racemase is also upregulated in cancer cells, where it is aggressively notably in prostate cancer where levels of the enzyme are actually a clinical marker of disease activity (Kong et al., 2020; Kong et al., 2021). Tuberculosis strains also exploit the racemase to expand their pathogenic niche and repertoire (Lu et al., 2015).

### Pseudo-bicharlity: Z-DNA: homochiral (helical) heterochirality

Many organisms, using canonical R-DNA/RNA, enrich (left-handed helix) Z-DNA/RNA, which is typically a feature of heterochiral L-DNA/RNA. It is termed here pseudo-bicharilty or pseudo ambidexterity due to the fact that residues are homochiral, however the macro-structure adopts a macro-blueprint associated with heterochiral residues, namely, a left-handed helix. Adoption of Z-DNA *in lieu* of the orthodox B-DNA is a rudimentary maneuver to evade nucleases and host immunity. Indeed, the very first virulent/parasitic lengths of DNA to invade the host genomes, *Alu*, may have exploited this B- to Z-DNA translation to promote their survival. *Alu* are mobile self-replicating regions of DNA (Herbert, 2024). A superfamily of immune mediators contains a Zα domain which binds Z-DNA with very high affinity. The first of these identified was ADAR1 (adenosine deaminase acting on RNA 1) (Tang, 2022). ADAR1 converts Adenosine to Inosine. This is recognized as Guanine by the host. Hence, there is an A to G base change, generating an untranslatable non-sense code.

Similarly, to generate the biofilm, bacteria specifically enrich and extrude Z-DNA or left-handed helical DNA. This is resistant to host DNA-ases (York, 2022; Buzzo et al., 2021; Gallucci, 2024). Hence, it is an interesting speculation to consider if mirror microbes would therefore generate a B-DNA biofilm. This would be exceptionally vulnerable to host DNA degradation enzymes. Further, it means indigenous Z-DNA is far from being an immunogenic. It is actually implicated in autoimmunity (Klein et al., 2025).

This discussion on the presence of metabolic dual chirality pre-existent in nature is critically important for a number of reasons, one of which we engage with here. It dismantles the theoretical obstacle to ambidextrous life: that such an existence is energetically unfavorable due to the huge cellular resources necessary for two copies of each enzyme, one for each chirality. We see here, already present in nature, enzymes that bind both enantiomers of the most emphatically chirality polymers, namely, DNA. Hence, even in the absence of dual chiral enzymes, a single or a few promiscuous racemases could generate all necessary substrates. Further, microbiota expend vast amounts of energy on immune evasion in all its guises and faces (de Jong et al., 2019; Howden et al., 2023). The need for these instruments of the immune arsenal could be circumvented by rendering an ambi-chiral invader immunological invisible with an astute chiro-deviant cloak, with alternating chirality residues, for example. This would eliminate the need for microorganisms to expend energy on producing multiple anti-immune factors. Metabolic ambidexterity could conceivably both increase the range of accessible trophic substrates but also conserve energy by eliminating the need for the myriad counter-immune agents and immune-modulators essential to the survival of homochiral species.

## Cancer conundrum: heterochiral microcosm

A common apothegm is fact is stranger than fiction. The same pertains here. While nature dabbles with but never fully unleashes its unfettered potential, cancer fully engages and wields heterodox chirality. Cancer cells show remarkable metabolic plasticity. Indeed, they show chiral “ambidexterity”. Cancer cells have been shown to actively uptake L-glucose in addition to D-glucose (Ono et al., 2020; Anastasiou et al., 2021). Neoplasia also frequently use and indeed concentrate counter chiral D-amino acids as metabolites and structural components in proteins (Murtas and Pollegioni, 2023). This has been implicated in tumor aggressiveness and resistance to chemotherapy (Uifălean et al., 2025). The situation also changes somewhat if tumors are exposed to ambidextrous or anti-lateralized microbiota. The tumor microbiome is increasingly found to be necessary for tumor survival, propagation, and dissemination (Cao Y. et al., 2024; Schorr et al., 2023; Jiang et al., 2024). Further, there may be metabolic synchronization with cancer and counter-chiral microbiomes. If, as speculated earlier, heterochiral ATP synthase will hydrolyze ATP to ADP and phosphate on proton flux, chiro-divergent biota may be entirely dependent on glycolysis for ATP generation, as is the case with many types of tumor. Cancer cells, even in the presence of abundant oxygen, satisfy their enthalpic demands with glycolysis alone. This is aerobic glycolysis or the Warburg effect (Thompson et al., 2023). Glycolysis is much more rapid than the Krebs Cycle and electron transport chain. Aerobic glycolysis thereby serves as a strategy to rapidly deplete the tumor milieu of metabolites, rendering it hostile for invading immunocytes, some of which then become effete. The effect would be amplified by heterochiral microbes, also exploiting glycolysis to sequester resources. The tumor and microbe could collaborate on a number of levels to subdue, evade, and thwart host immunity. Chiral plastic cancer with “ambidextrous” or even mirror pathogens threaten to create a devastating counter-chiral microcosm, invisible and invulnerable to host immune attack, yet able to deploy immune checkpoint machinery and avaricious in consumption of host normo-chiral resources while nourished by mirror pathogens’ hetero-chiral nutrients.

## Chiral caution and enantiomeric accountability

Given the potential evolutionary survival advantage enjoyed by ambidextrous organisms, as exemplified by cancer, some foresight and planning is required for their eventuality. The UK government on 12 June 2025 released guidance and research on the hazards, merits, and demerits of mirror life forms specifically. The conclusions were that the benefits outweigh the risks, but tangible risks exist and safeguards must be engaged. I share this sentiment with two critical caveats. Firstly, as suggested, the main risks and greatest benefits come from ambidextrous chiral lifeforms, particularly those capable of both hetero- and homo-chiral activity. Secondly, as is the case with human hubris, we have all assumed the counter-chiral and dual chirality microbiota could only result from human ingenuity. However, as demonstrated in the foregoing discussion, a high degree of biochemical ambidexterity already exists in nature, even to the point of bi-chiral DNA binding enzymes and protein-folding chaperone proteins–the fundamental infrastructure of life. This widely opens the door to the “natural” or evolutionary provenance of such organisms. Hence, in addition to reflective practices on human endeavors, there is also need for surveillance in nature itself. If there were pathogens now that were exploiting counter-enantiomeric pathways, not only would this be overlooked by human immunity, it might also be missed by human cognition, as we would not be expecting nor actively pursuing this putative pathogenic route.

Research in this arena, in the early years, will require strict supervision and accountability. It is equally important to review and expeditiously report progress in this arena. There may be a need for registration and licensing. The UK Human Embryology and Fertilisation Authority oversee research on embryos in the United Kingdom. The International Society for Stem Cell Research (https://www.isscr.org/) and National Institute for Health (NIH) have a comparable international authority and role but not legal force (Lo et al., 2010). However, the question is whether an analogous body in the arena of ambidextrous biology would in itself be sufficient. There are moves to extend the embryo research limits from 14 days to 28 days (https://hdbi.org/public-dialogue), abandoning this threshold in certain circumstances. This is notwithstanding the biological consensus that life begins at conception (Jacobs, 2021). This raises the enduring question of whether human ingenuity can provide sufficient safeguards in areas of high-risk but highly promising research.

Consideration must be given to universal contingency plans. The Classical Jurassic Park Contingency Plan would see all engineered lifeforms to be auxotrophs whose survival is critically contingent upon a factor provided to the culture medium. (In Jurassic Park, the dinosaurs needed high-dose lysine in their diet and thus theoretically could not survive outside of the park.) While chiral chimeric microbes may lose such dependence, as the dinosaurs in Jurassic Park, the advantage of some for trophic restraint is two-fold. Firstly, it provides a ring-fence of protection rather than completely dysregulated growth. Even if they were to circumvent this restraint, it would be a starting point to detect a vulnerability to an otherwise potentially invulnerable species. The narrow biochemical repertoire by which such dual-chiral organisms unfetter themselves from auxotrophic regulation could then be targeted.

Consideration must be given to the feasibility and efficacy of any regulatory framework. Ambidexterity is a feature of extant species. Hence, it is impossible to proscribe research in biological paradigms that already occur in nature. Any research in mirror and ambidextrous microbiomes could be justified under the pretext that it merely reflects physiological processes. The raises fascinating considerations. In the same manner that uranium enrichment to a certain percentile is indicative of nuclear armoury, it is the threshold per centage of biochemical ambidextrous enrichment that can be seen as sinister and indicative of nefarious rather that novelty-seeking research practices. However, the existence of cancer itself undermines this approach. We have already seen how cancer cells intentionally enrich hetero-chiral substrates and racemases above that seen physiologically with α-Methylacyl-CoA racemase actually in clinical use as screening tool for prostate cancer. Pro-tumor cells lines and even strains of research animals with specific proclivities to various cancers are frequently used in clinical research (De Vleeschauwer et al., 2024). Indeed, in the current climate, research investigating the effect enriching heterochiral bio-chemical, enzymes, and racemases on such cells and species would not be considered unethical or hazardous. Granted few cancers, if any, are communicable. Such heterochiral or ambi-chiral enrichment in microbiota would clearly be more controversial.

## Iatrogenic solutions to subversive stereo-isomeric species

Given the innate challenges in regulation, the fallibility of contingency measures, the prevalence of metabolic ambidexterity, and the distinct possibility that dual chirality species may emerge organically, it is necessary to consider possible means of combatting such life-forms. Such organisms are undoubtedly formidable. However, they must not be invariably seen as our microbial Kobayashi Maru. To do so would be to underestimate human ingenuity. Ironically, we have recently developed the biochemical armamentarium to counter counter-chiral life.

### Sequence and subdue

As has previously been observed, heterochiral peptides exhibit immunogenicity. One potential therapy against contralateral life would be to sequence a peptide segment and use this to “prime” host immunity. The most conspicuous motifs both sterically and immunologically could be selected. Artificial Intelligence and, in particular, AIphaFold could facilitate elucidation of the 3-dimensional configuration of peptides (Jumper et al., 2021). The sophistication of immunity means that this is not necessarily an entirely anodyne intervention. IgG3 has shown bi-chiral avidity and hence may cross-react with host epitopes with similar but contra-chiral sequence. This is amplified by the Network Theory paradigm. This is the phenomenon where antibodies to antibodies’ variable moieties (anti-idiotype antibodies) function to temper immune responses but may have a pathogenic effector function. Hence antibodies-to-antibodies to non-canonical chiral motifs may attack orthodox chiral signatures (Murphy and Longo, 2022). In this we return again to the phenomenon whereby the comprehensiveness and sheer ineluctability of the immune system means its response to neo-antigens is uncertain.

The second hazard relates to the potency of the immunity. “Immunization with heterochiral oligo-peptides” would suddenly and dramatically uncloak a microbe that was hitherto immunologically invisible. This could elicit an over-zealous and over-exuberant immune response that would compromise the host. A similar patho-immunological paradigm is observed with the Guinea worm (*Dracunculus medinensis*). The worm is typically extracted through the skin, meticulously and delicately, to avoid death of the worm while remnants reside within the tissue. The reason for this perfectly demonstrates the risk of suddenly awakening host immunity to a counter-chiral pathogen. While alive, the Guinea worm produces factors that render it largely immunologically inert. In particular, it suppresses INF-ɣ and specific IgE responses. The helminth even generates morphine and morphine analogues to pacify the host (Knopp et al., 2008; Simonetti et al., 2023). However, if extracted too vigorously, the head is separated from the remainder of the body. In its demise the body loses its immunological cloak. A microbe centimeters in length is then suddenly and precipitously exposed and unmasked to the full force of immunity, resulting in an immunological tempest that may eradicate the host.

To mitigate the risk of harm from over-zealous immunity, specific facets of the immune system could be selectively exploited, for example, by generating and creating murine chimeric antibodies by exposing mice rather than human hosts to the chiro-aberrant heptads/oligopeptides.

If the obverse is the case and oligopeptide heptapeptide from the mirror or chiral chimeric life remains, hypo-immunogenic adjuvants can be appended to the molecule to potentiate immunity (Zhao et al., 2023).

### Viral vector redux

Viral vectors are an ingenious means of immunization. Their use to combat mirror life is not impossible but faces a number of challenges which must be addressed before this paradigm can be deployed. Firstly, the human hosts simply lack the substrates to create heterochiral protein or peptides. Possible solutions include using the genome of the unconventional chiral species essentially as a live attenuated vaccine in a viral envelope. More biochemically frugal methods would involve incorporating racemases and exploiting chaperones in nature which have been shown to be ambidextrous in protein affinity. Transcription, translation, and polymerization with counter-chiral amino acids would still remain challenges to definitively resolve and would require co-opting ambidextrous machinery from other species. Given the unchartered territory of counter-chiral life vaccination, viral vector vaccines not associated with vaccine induced thrombotic thrombocytopenia (VITT) should be used (Benemei et al., 2025), namely, the RNA vaccines.

### Chiral chimera-targeted CAR-T

Chimeric Antigen Receptor–T cell Therapy is potentially the most sophisticated tool in any fight against species exhibiting chiral aberrant phenotypes. T-cells can be honed to target immunogenic peptides from heterochiral species (Peche and Gottschalk, 2025).

### Achiral answers

Subcellular functions are orchestrated by bodies known as condensates, which are phases of matter or aggregates of cellular effectors such as ribosomes and enzymes. Disrupting condensates arrests cellular function (Author Anonymous, 2025). A so called “kill switch” has been devised, an oligopeptide, which disrupts condensates and causes dense cessation of cellular function, including at the nuclear level (Zhang et al., 2025). It is likely that their function is not enantiomer-dependent and may function on trans-lateralized organisms and ambidextrous species. Even if it was chiro-dependent, such kill switch proteins are as diminutive as 17 residues and thus potentially synthesizable in any chirality.

## Conclusion

The discussion of mirror microbes is necessary and essential, but it must reflect the reality that life itself is exquisitely dependent on sequentially contingent events. Very small changes in events, especially at the prebiotic phase, would have made life impossible, dramatically different to that which is observed today, or perpetually confined to the infundibula stages. For example, life may not have emerged at all had the Earth’s oceans not been depleted of deuterium early in prebiotic evolution (Pope et al., 2012; Qu et al., 2024). The recent recovery of organic molecules from the Bennu meteorite, which show equal amounts of both enantiomers, suggest that during the first steps of evolution, one chirality was preferred over the other (Witze, 2025). In summary, to suggest, after billions of years, the emergence of heterochiral species, which were essentially the first phenotypic choice to be deselected by natural selection, would now be monarch of the microbiota is unconventional. However, it is time to afford greater attention to the exploitation of chiral plasticity by tumor cells to circumvent immunity and subdue hosts and in nature. In many micro-organisms there exists some degree of metabolic ambidexterity. This may spawn dual chirality species, or they may result from human investigation. Such bio-chiral manipulation is difficult to regulate given that nature itself does not exhibit chiral exclusivity but rather enantiomeric diversification. In this light, strategies must be devised for the emergence of such biota. The timely advent of vector vaccines and CAR-T combined with the recent epiphany on the role of condensates could prove invaluable tools in such an arsenal.

## Data Availability

The original contributions presented in the study are included in the article/supplementary material; further inquiries can be directed to the corresponding author.
